# An Overview of Ingredients Used for Plant-Based Meat Analogue Production and Their Influence on Structural and Textural Properties of the Final Product

**DOI:** 10.3390/gels9120921

**Published:** 2023-11-22

**Authors:** Maja Benković, Ana Jurinjak Tušek, Tea Sokač Cvetnić, Tamara Jurina, Davor Valinger, Jasenka Gajdoš Kljusurić

**Affiliations:** Faculty of Food Technology and Biotechnology, University of Zagreb, Pierottijeva 6, 10000 Zagreb, Croatia; ana.tusek.jurinjak@pbf.unizg.hr (A.J.T.); tsokac@pbf.hr (T.S.C.); tamara.jurina@pbf.unizg.hr (T.J.); davor.valinger@pbf.unizg.hr (D.V.); jasenka.gajdos@pbf.unizg.hr (J.G.K.)

**Keywords:** plant-based food, protein, texture, 3D print, fats, additives, spices

## Abstract

Plant-based meat analogues are food products made from vegetarian or vegan ingredients that are intended to mimic taste, texture and appearance of meat. They are becoming increasingly popular as people look for more sustainable and healthy protein sources. Furthermore, plant-based foods are marketed as foods with a low carbon footprint and represent a contribution of the consumers and the food industry to a cleaner and a climate-change-free Earth. Production processes of plant-based meat analogues often include technologies such as 3D printing, extrusion or shear cell where the ingredients have to be carefully picked because of their influence on structural and textural properties of the final product, and, in consequence, consumer perception and acceptance of the plant-based product. This review paper gives an extensive overview of meat analogue components, which affect the texture and the structure of the final product, discusses the complex interaction of those ingredients and reflects on numerous studies that have been performed in that area, but also emphasizes the need for future research and optimization of the mixture used in plant-based meat analogue production, as well as for optimization of the production process.

## 1. Introduction

Climate change and the sustainability of the production process are some of the most pressing problems in food production. The impact of food production and type of diet on climate change is measured by the carbon footprint, and, according to the research available so far, the change in the eating habits of consumers from the predominant Western type of diet to a Mediterranean type, vegetarian or vegan diet also contributes to the carbon footprint reduction by minimizing greenhouse gas emissions and reduction in water consumption required for production [1,2]. The earlier mentioned is one of the reasons why consumers are continuously turning to healthier alternatives to the Western diet. Accordingly, the demand for vegetarian and vegan food products is growing, as evidenced by the fact that the size of the vegan food market was estimated at USD 19.7 billion dollars in 2020, and is expected to reach a market worth of USD 36.3 billion by 2030 [3]. In accordance with the increased demands for vegan food, the technology for the production of such food is also being developed, which includes novel drying processes, protein extraction, protein purification, microencapsulation, 3D printing and more [4,5,6]. One of the new technologies whose application is increasingly common in the production of vegan food is additive technology, or 3D printing technology. Additive technologies are characterized by advantages that include the possibility of creating complex shapes, the possibility of regulating texture and color, the protection of thermolabile ingredients and the possibility of creating personalized products [7]. Additive technology involves construction of solid geometries in multiple layers, which are then connected by chemical reactions or phase transition processes [8]. Some of the most commonly used 3D printing technologies are extrusion printing, ink jet printing, bio printing and printing by deposition and bonding powder (binder jetting) [7,9]. Briefly, the extrusion process uses semi-solid pastes, which are then squeezed out of syringes with different nozzle diameters and geometries to form a specific shape, previously defined using a 3D modeling software (CAD software). An ink jet requires liquid materials with a significantly lower viscosity in comparison to pastes used for extrusion, which are then sprayed or dripped to a desired form [9]. Binder jetting is a specific process where powders are used as raw materials to form final products, while bioprinting is usually connected to printing forms comprised of living tissues. According to literature data, in the food industry, 3D printing is applied to cereal products, confectionery, pasta, cheese, fruit products, surimi, etc. [8,10,11,12,13].

In the production of plant-based meat analogues, besides 3D printing, other technologies (e.g., shear cell, high- or low-moisture extrusion and others) can also be applied to produce a product that resembles meat, but is completely plant-based. The most common type of technology used is extrusion printing, where it is of importance to adjust and optimize the composition of the printing mixture so that, first of all, printing is possible, and then so that the said product has satisfactory nutritional, sensorial and textural properties, which are of primary importance to the end consumer. Plant-based meat analogues are often characterized as mixed gel systems produced with the texturization of various plant-based ingredients (e.g., cereals, legumes, algae, fungi) [14]. The most common ingredients in plant-based meat alternatives include soy, legumes (e.g., chickpeas, lentils, peas), grain proteins (gluten from rice, oats, barley or wheat), colorings (cumin, carotene, lycopene, beetroot juice) and flavorings (spices, herbs, yeast extract, paprika, sugar, mushrooms). In addition to the above, to ensure the texture and properties similar to meat, oils (sunflower, coconut, palm, etc.) are used, as well as various “structural” ingredients responsible for the connection and stability of the shape (carrageenan, starch, cellulose, etc.). Furthermore, the mentioned mixtures can be enriched with various vitamins and other bioactive ingredients [15].

This review provides an insight into the most common ingredients used for production of plant-based products, with a special focus on their structure and the ability of the ingredients to affect and regulate the texture, structure and nutritional properties of the final product. It is divided in sections, which focus on specific groups of ingredients: proteins and fats, ingredients responsible for maintaining acceptable texture of the products; spices, colorings and functional ingredients, which can act as stabilizers; and enrichment agents.

## 2. Proteins

**Figure 1:**
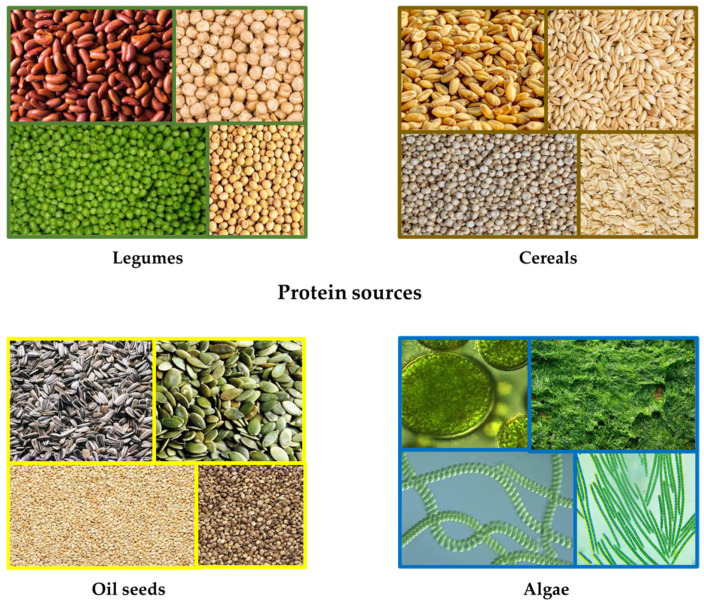
Protein sources used in plant-based meat analogue production.

Proteins are a group of macronutrients, which are considered to play a major role in building muscles and tissues and are crucial in maintaining proper functions of the human body. Humans mostly ingest proteins of an animal origin, but the ever-growing shift towards healthier alternatives has put the plant-based protein sources in perspective. Figure 1 depicts some of the plant-based protein sources.

As seen in Figure 1, based on their origin, plant-based protein sources can be divided into several groups: (1) legumes (soy, pea, chickpea, lentils, beans, peanuts); (2) cereals and pseudocereals (wheat, corn, oats, quinoa, amaranth, buckwheat); (3) oil seeds (hemp, sunflower, rapeseed, sesame); and (4) algae. While some of the mentioned groups (e.g., legumes and cereals) contain proteins that are able to affect the structure of meat analogues and can therefore be used to produce meat analogues of a desired texture and mouthfeel [16], others are primarily used as good sources of amino acids and bioactive peptides [17,18]. Furthermore, besides proteins, seeds can also act as sources of fats necessary to ensure texture, flavor, mouthfeel and juiciness, but also as sources of fat-soluble vitamins and fatty acids [15]. 

### 2.1. Legumes as Protein Sources

Legumes are plants belonging to the *Fabaceae* family and are characterized by a high nutritional value. They are rich sources of proteins (20–30%), fiber, micronutrients and antioxidants. Soy, lentils, pea, chickpea, beans and peanuts are some of the representatives of the group, which will be discussed in this subsection.

#### 2.1.1. Soy

Soy (*Glycine max*) is a legume widely present in the East Asian part of the world. It is an annual species that is mostly cultivated because the bean of the plant is edible and has a dense nutritional composition—it is rich in proteins, fat, carbohydrates, fibers, micronutrients and vitamins [19]. In general, soybeans contain around 35–40% protein, 20% lipids, 9% dietary fiber and 8.5% moisture (d.w.), with an emphasis that this composition is highly dependent on the cultivar, location and climate conditions [20]. Major beneficial constituents of soy and their functional properties are shown in Table 1.

Soybean can be eaten as is or processed and used in different foods such as milk substitutes, meat substitutes, sauces and fermented foods. In terms of technological suitability, soy proteins are of great value to the food industry because its proteins can enhance water holding capacity, bind fat and have gelling properties [9]. Although it is considered as a great source of protein for vegetarians, vegans and flexitarians, its use is also followed by a controversy connected to genetically modified crops [24]. Other problems with its use also include high allergenicity of soy.

Soy flour is produced by grinding soy flakes, which can be full-fat or defatted, and is considered to be the least processed and the cheapest form of soy, which contains about 50% protein. The concentrate contains approximately 70% protein, and it is made with alcohol extraction, while the isolate is produced with alcoholic extraction combined with acidic pH precipitation, which raises the protein content to 90% [25]. Soy proteins can be used directly in meat analogue mixtures, or they can undergo texturization [26]. Texturization is a process in which proteins can be converted to a fibrous structure which resembles meat, and it can include processes such as extrusion cooking, electrospinning, freezing, shear cell or Couette Cell technology [27,28,29,30,31]. Texturization usually results in soy proteins that provide better mouthfeel, chewiness and hardness to the final plant-based product [15].

Available literature containing examples of soy protein use in the development of plant-based food is numerous, since this area of food production is still in development. Focus of the studies is based either on regulation of structural properties of products, nutritional composition (design of tailor-made foods) or consumer perceptions of the plant-based meat analogue products. As stated before, the focus of this review is primarily on textural and structural properties, and the following paragraph presents some of the newest examples of literature-available data. Samard and Ryu [32] have researched the differences in physicochemical and functional characteristics of textured isolated proteins originating from soy, mung bean, peanut, pea and wheat, and concluded that soy proteins exhibited high textural properties and a more sponge-like structure in comparison to other proteins. However, they also emphasized a change in the amino acid profile of proteins after extrusion and a drawback of soy proteins—the lowest essential amino acid content in comparison to other proteins. Carranza et al. [33] investigated the suitability of a soy protein isolate in different concentrations (20, 25 and 30%) to produce soy-based food for people with swallowing disorders. They concluded that the addition of 25% of a soy protein isolate to the dough resulted in doughs with a shear thinning behavior, which is favorable for 3D extrusion printing. After printing, the doughs maintained the desired shape, and the infill rate had a significant effect on hardness, gumminess and chewiness of the hydrated samples. Taghian Dinani et al. [34] investigated the influence of hydrocolloids in combination with soy protein isolates on the texture of soy protein blends. They concluded that a soy protein isolate, as a control sample without hydrocolloid addition, exhibits a homogenous gel structure, which is not beneficial in meat analogue production, and proposed the use of soy protein in combination with hydrocolloids to obtain a fibrous texture, which is preferred for meat analogues. A similar conclusion was also reached in a study by Mao et al.—a soy protein isolate had a compact gel structure after extrusion [35]. Chantanuson et al. [29] studied the behavior of soy proteins in gel formation during freezing and concluded that the densest layered structure with high porosity and anisotropy was obtained when soy flour with a solids content of 10% was used for gel formation. Two studies by Shahbazi et al. revealed an in-depth analysis of soy-protein-based 3D ink formation—firstly, it was concluded that the network structure and texture of soy-based inks can be modified by the use of fat substitutes in the form of hydrophobically modified biosurfactants [36], and that the incorporation of biosurfactants improved the fibrous degree of the 3D printed structures [37]. Furthermore, Schreuders et al. [38] concluded that soy protein blends resulted in mechanically stronger materials in comparison to pea protein blends, but emphasize that this effect is dependent on the processing conditions—in this case, on shearing temperature. They explain their finding in relation to the difference in the microstructure of the blends (alignment of the protein domains and entrapment of air bubbles in the structure).

In general, the conclusion that can be drawn from all of the above-mentioned facts and research overview is that soy proteins are one of the most widely used and researched components of plant-based meat analogues, owing their use to good nutritional composition and the ability to regulate textural properties of the final product. However, there is still more research that can be performed in this area—e.g., which concentrations of soy proteins to use for a specific application; which additives, colorings, spices and structural ingredients; and, in the end, which processing technique (freezing, extrusion, shear) and at which processing conditions. All of those questions need to be answered for each specific application. Also, research needs to be conducted on specific protein interactions during the production process, on ways to reduce allergenicity of soy and to change consumer perceptions of soy as a genetically modified crop.

#### 2.1.2. Pea, Chickpea, Lentils, Other Beans and Peanuts

Other plants from the *Fabaceae* family are peas, which can include different varieties such as garden or field peas. According to Kumari and Deka [39], green peas contain 20–50 g of starch, 17–22 g of carbohydrates, 14–26 g of dietary fiber, 6.2–6.5 g of protein, 0.4 g of fat and 1.0 g/100 g of ash with a markable amount of vitamins and minerals. They are rich in lysine and threonine, have a low glycemic index and have many beneficial effects in helping to prevent and treat cancer [24]. From a technological point of view, pea proteins are important because they can form gels and emulsions and stabilize foams [16]. However, in comparison to soy proteins, their gelling capacity is much lower, so the mixtures have to be supplemented with different salts or structural agents to result in gels with the same textural properties as those made from soy proteins [9,25]. Higher gelling capacity of soy in comparison to pea can be explained by soy having the constituents that increase its water-holding, gelling and fat-absorbing properties [9] and by a higher protein unfolding grade of soy proteins during processing [40]. In spite of lower gelling properties, pea proteins are beneficial because they are more accessible—peas can be grown in moderate climates, are not as allergenic as soy and they do not have the GMO stigma attached to them. Similar to soy, pea proteins can be used in the form of isolates or can be textured to obtain fibrous structures. Some of the latest studies on the applicability of pea protein for meat analogues and their beneficial influences on textural properties are shown in Table 2.

Besides the above listed, an interesting study was conducted to assess the environmental impact of pea-protein-based meat alternatives—the path from pea cultivation, protein processing and its utilization into pea sausages in Sweden was followed. The authors concluded that the most contributing stages of the whole process were cultivation of peas and production of sausages (0.23 kg of CO_2_ eq per kg of sausage for cultivation and 0.52 kg of CO_2_ eq per kg of sausage for production) [46]. In comparison, only the production phase of a meat-containing product—bean and pork stew—results in 2.23 kg of CO_2_ eq per kg of stew, which is considerably higher [47].

To summarize, pea proteins appear to be a promising alternative to soy protein, mostly due to their better market availability and the lack of the GMO stigma. However, in comparison to soy, texture regulating properties seem to be less expressed, and are therefore often used in combination with other protein sources and structural ingredients. Furthermore, there is a need for better structural identification of proteins, since some studies concluded that the purified forms have better gelling abilities. Also, the structural properties depend significantly on processing conditions: moisture content, temperature and the type of process used to produce pea-protein-based products.

A short overview of textural properties of chickpeas, lentils, beans and peanuts is shown in Table 3. The listed protein sources and properties are further discussed in more detail in the text that follows.

Chickpea (*Cicer arietinum*) has been known and used in food products since ancient times, but it is now moving through its novel boost in popularity since the needs and the requests of consumers are slowly but steadily shifting towards plant-based foods. Also, its benefits include no allergenicity and no phytoestrogens. Chickpea proteins can be used in a form of a powdered isolate, which contains all essential amino acids and has a high amino acid score [61]. It is known to be rich in proteins, which include globulin, albumin, prolamin and gluten [62]. The biggest issue for the chickpea proteins is its low water solubility and the dependence of its functional properties (gelling behavior, emulsification and foaming ability) on the pH value of the medium, which, in consequence, renders its applicability in food production. However, these obstacles can be overcome with protein modifications using thermal, mechanical or enzymatic treatment [61]. Kaur and Singh [48] investigated functional and thermal properties of Indian chickpeas and determined that the foaming capacity values ranged from 30.4 to 44.3%, which is significantly lower than the foaming capacity of soy protein isolates (235%), due to a high share of globulins, which are not prone to surface denaturation. However, the stability of foams produced using chickpea proteins was rather high (94.7% after 120 min of storage). Furthermore, the gelling capacity was determined to range from 14 to 18%, which is in range with that of soy protein isolates (16–17%). According to Kumar et al. [49], chickpea protein isolates have higher oil and water binding capacities in comparison to soy protein isolates and have an additional benefit—they can be used for improving the color of meat analogues because of the presence of carotenoids.

Lentils (*Vicia lens* or *Lens culinaris*) are also an ancient crop marketed as green or red lentils. They are a rich source of protein, dietary fiber, complex carbohydrates, iron, zinc and B vitamins and are high in phenolic compounds [63]. Lentil seeds contain an average of 26% of protein, with the protein content varying depending on the geographical origin and climate. In most part, proteins are globulins and albumins, with glutelins and prolamines present in lower amounts in some cultivars [64,65]. Also, there are numerous studies describing antioxidant, antihypertensive and antifungal properties of lentils [63,66,67]. Functional properties of lentil proteins, which are beneficial to the food industry and the production of plant-based foods, are mostly researched in terms of determining their solubility, foaming capacity, foam stability, emulsifying properties and water- and oil-absorption capacity [64]. Tang et al. [50] researched the gelling properties of lentil proteins at different pH values and concluded that their gelling behavior is highly pH-dependent: the best gel formation was achieved at pH = 3.0, with the values of the storage modulus being comparable to those of whey proteins, which indicated the possibility of lentil protein use in foods with specific pH values. Based on literature data, change in pH changes the net charge of the protein, which, in consequence, changes its gelling behavior [68]. According to a study by Jo et al. [69], lower pH (pH = 2) causes the formation of a stronger network because of linear fine protein structure formation, which is capable of building a more interconnected network. A very detailed and extensive study was performed by Shrestha et al. [51] on the rheological and textural properties of heat-induced gels from pulses. They discovered that lentil protein isolates had lower water holding capacities in comparison to soy, but higher in comparison to pea protein isolates. On the other hand, the oil holding and the foaming capacity was comparable to that of soy and pea proteins, and all of the samples exhibited excellent potential as emulsifiers. Guidi et al. [52] noticed that lentil-based gels had higher gel strength compared to other gels analyzed in their study (hemp and pea), but also noticed that the gel strength rises if binary mixtures of those proteins are produced. In general, lentil proteins show promising properties for their application in plant-based products, but, in comparison to soy, they still have less pronounced texturization possibilities. Another problem evident with lentils is the fact that they contain a great number of compounds that are considered to be antinutrients [70], which need to be modulated or removed prior to their application in cooking or plant-based food production.

Among beans most often mentioned as promising ingredients in meat analogues are faba and mung bean proteins. Faba bean (*Vicia faba* L.) contains 29% and has higher antioxidant capacity than peas and soy [62]. Similar to other legumes, faba bean proteins have good emulsifying and foaming properties, but are much lower in comparison to soy. Interestingly, there is a lot of literature data available on faba bean proteins, much more than for chickpea or lentil proteins, which confirms their potential for the use in food applications. Saldanha do Carmo et al. [55] explored the suitability of faba bean protein for the low-moisture-extrusion process. A twin-screw extruder was used to perform the low-moisture extrusion at 900 rpm and temperatures ranging from 40 to 130 °C. It was concluded that the extrusion-functionalized faba bean proteins had significantly higher sectional expansion index, water holding capacity, water solubility index, browning index and juiciness. They also emphasized that the desired physicochemical and technological properties of the faba bean proteins are highly dependent on the process parameters of the extrusion process. Hu et al. [53] investigated the properties of faba bean protein emulsion gels for 3D food printing and concluded that the gels formed with faba bean protein isolates can be finely tuned for 3D printing with a heat treatment (90 °C for 30 min), which significantly raises the gel strength. Furthermore, Ferawati et al. [54] managed to produce fibrous layered structures from faba bean protein isolates with high-moisture extrusion, which are suitable for use in plant-based meat analogue production. Mung beans (*Vigna radiata* L.), on the other hand, are characterized by 25–28% protein content, only 1–2% fat and significant amounts of amino acids: proline, glutamic acid, arginine, leucine and phenylalanine [24]. Physicochemical and technological properties of mung bean proteins are also highly dependent on the form in which they are used—Yang et al. [56] concluded that mung bean globulins form weak interfaces and unstable forms, while mung bean albumins form stiff interfaces and stable foams. Dry fractionated mung bean applicability for meat analogue production was tested by De Angelis et al. [71], who concluded that, for lentils and faba beans, temperature is the most important process parameter, which influences textural properties of the extrudate, and have proven that the mung bean proteins can be used in meat analogue applications.

Peanut is a widely available legume, which is known for its good nutritional properties. Although the main ingredient of peanuts is lipids, which make up about 50% *w*/*w* of the nut, they are also rich in proteins (approximately 25%) [62]. Peanuts also exhibit beneficial effects and help in prevention of diabetes and cardiovascular diseases [72,73]. Studies have shown that peanut proteins do not form gels at low temperatures. Gel formation occurs after a thermal or enzymatic treatment [74,75]. The high-moisture extrusion process of peanut protein biomass was explored by Zhang et al. [58] with the findings that the proteins undergo structural changes in the extruder, which are responsible for the formation of a meat-like fibrous structure. They also identified that arachin was the main protein in fibrous structure formation. There are also studies in which peanut proteins are combined with other structural ingredients—Lin et al. [59] concluded that peanut proteins with the addition of carrageenan and gellan gum result in gels with increased storage modulus and fracture stress, while Guo et al. [60] combined transglutaminase and peanut proteins in a high-temperature pressure cooking process to obtain tofu with improved textural properties. In comparison to other legumes, peanut protein has less pronounced technological benefits, but can be modified to improve its drawbacks. Another problem connected with peanut utilization is its high allergenic potential, either consumed by itself or in combination with other foods or allergens where cross-reactions can occur. A recently published review paper by Haidar et al. [76] offers several technological solutions to reduce allergenicity of peanuts, which include processing at high temperatures (e.g., boiling, baking, frying) or the use of ultrasound and high pressure (autoclaving).

### 2.2. Cereals and Pseudocereals as Protein Sources

#### 2.2.1. Cereals

Cereals are one of the most widely spread and readily available crops, which makes them interesting not only in basic food production (bread, cookies, pasta, etc.), but also as a valuable ingredient of other food products in which textural properties need to be regulated. They can be used in a form of flour (maize, wheat, rye), flakes (oat) or seeds (rice, maize) [77]. Although cereals are higher in carbohydrates than in proteins, proteins present in them are technologically interesting because of the viscoelastic structural network, which binds the components and helps to provide consistency and fibrous texture, which are mandatory in the production of plant-based meat analogues [78]. Wheat, maize, rice and oats are some of the examples of cereals used in plant-based meat products. Table 4 shows the proximate protein content of those cereals.

Types of proteins present in cereals include albumins, globulins, gliadins and glutelins, but, by far, the most important component is gluten, which is obtained as a byproduct of wheat flour wet processing. Gluten has beneficial viscoelastic properties and enables regulation of textural properties of plant-based meat analogues, and, at the same time, it is an economically feasible ingredient [24], is FDA approved and has a GRAS status [77]. On the other hand, gluten intolerance is also a known issue today, and there are a lot of literature examples where gluten-free blends are made to alleviate the symptoms of people suffering from coeliac disease [79,80].

A study by Wang and Liu [81] investigated the suitability of soy, gluten and cocoa butter mixtures for 3D printing of meat analogues and concluded that, in order to obtain a gel with desirable textural properties for a 3D print, an addition of other structural ingredients (cocoa butter, starch, Tween-80, sodium alginate or shiitake mushroom powder) is required. Interestingly, oat is often present in the most novel studies about its applicability to form structured gels. Nikinmaa et al. [82] extruded mixtures of rice and oat flour at different temperatures (120–160 °C) and moisture contents (14.5–20.6 g/100 g) and concluded that the best structure of the extrudates was achieved at low-moisture/high-temperature conditions. An oat protein concentrate in combination with transglutaminase was explored by Pöri et al. [83], where the positive effect of transglutaminase was found to be significant on viscosity and tensile strength, enabling the formation of fibrous extrudates. Addition of an oat fiber concentrate to pea protein isolate mixtures for extrusion was found to reduce structural strength (chewiness and hardness) of the fibrous meat analogues, and higher concentrations of oat fiber led to a reduction in porosity and a lower water holding capacity of the meat analogue. This was an indication that oat can be used in meat analogue production, but only in a specific concentration range (30–50%) [84]. A combination of oat and pea protein blends for low-moisture extrusion was investigated by Kaleda et al. [85] and they concluded that the extruded product had good flavor and a fibrous structure. Some of the most novel research includes the application of cereal brans in the production of extruded meat alternatives. Rekola et al. [86] used oat and wheat bran in combination with a pea protein isolate to produce meat analogues and managed to produce an analogue with higher tensile and cutting strength, which is typical for a fiber-like meat structure. They also concluded that bran inclusion can lead to the improvement of textural properties of meat analogues.

#### 2.2.2. Pseudocereals

In review papers dealing with alternative plant-based protein sources, pseudocereals are often grouped together with cereals, mostly due to several very similar properties, although that classification is not entirely correct. Pseudocereals are a group of plants that, based on a review by Lingiardi et al. [87], form starchy seeds and are categorized as *Dicotyledonae*. However, from a nutritive and processing aspect, they are very much like cereals; the main fact that differs them from cereals is the presence of starch in perisperm, which is different to cereals where starch is stored in endosperm. Furthermore, they do not contain gluten [88]. Amaranth, quinoa and buckwheat belong to the pseudocereals group. According to Kurek et al., amaranth contains approximately 14% protein, while quinoa has 8% protein, with a high content of lysine, arginine and tryptophan. Quinoa contains all nine essential amino acids [19,24]. Quinoa protein isolates were studied by Mir et al. [89], with a conclusion that the isolates can be characterized by strong gelling characteristics. A review by Lingiardi et al. [87] describes quinoa protein isolates as promising emulsion stabilizers, with an emphasis on the fact that quinoa proteins do not require chemical modifications before their application in food products. On the other hand, studies by Cui et al. and a review by Wang et al. propose that the functional properties (e.g., gelling behavior) of quinoa proteins can be improved with physical, chemical and enzymatic modification [90,91]. Native amaranth proteins exhibit poor water solubility, and, therefore, processing is required to enhance their functional properties. Figueroa-Gonzales et al. [92] suggested pH and ultrasound treatments as means of improving their foaming capacity, stability and digestibility, but Graziano et al. [93] emphasize that the lack of a gluten network in pseudocereals still results in a major drawback in their technological utilization. They also mention another drawback—the presence of antinutrients and high levels of phenols, which makes them taste bitter. Buckwheat contains about 9–15% of proteins and it is a source of essential amino acids, but the lack of gluten-type proteins results in products with undesirable textural properties, leading to the necessity of its use in combination with other plant-based proteins, enzymes or salts [94].

### 2.3. Oil Seed Proteins

Oil seeds, as stated in their name, are primarily used as a source of oil. However, recently, in the quest for novel protein sources, they have been mentioned more often as high-quality protein sources, which are often texturized to obtain a desirable fibrous-like structure, which is beneficial for plant-based food production. Some of the examples include hemp, sunflower, rapeseed and sesame proteins. Some papers mention peanuts as a part of the oil seed proteins; however, in this review paper, peanuts have been mentioned in the legumes group, since their botanical classification puts them among legumes. A concise literature overview of the textural properties of some oil seed protein sources is summarized in Table 5.

Industrial hemp (*Cannabis sativa*) (Table 5) has been studied extensively in the past few years, and it is often described as a versatile crop because of its diverse application in a wide range of industries (food, textiles, paper, plastic, paint, feed). Hemp seeds are currently a topic of many studies, since they are considered to be an excellent source of vitamins, minerals, fibers, essential amino acids and fatty acids [102]. Sunflower meal/cake is often referred to as an alternative and sustainable source of proteins. The proximate composition of hemp seed depends on the cultivar and agronomic conditions, but, generally, it contains 25–35% fat, 20–25% protein, and 20–30% carbohydrates, which are mainly comprised of insoluble dietary fiber [97]. Majority of the proteins isolated from hemp seeds are globulins and albumins, with a secondary structure dominated by β-sheets (41–42%), which enable the formation of fibrous meat-like structures after extrusion [31]. Nasrollahzadeh et al. [95] explored the potential of hemp seed protein concentrates for production of high-moisture meat analogues. They discovered a significant contribution of disulfide bonds in hemp protein aggregates, which lead to the formation of anisotropic fibrous structures. Furthermore, they also concluded that dry fractionated hemp proteins contain high levels of phytic acid, and emphasized another role of hemp proteins, besides the structural one, as an important contributor to the daily intake of phytic acid. Zahari et al. [96] produced high-moisture meat analogues with extrusion using a hempseed protein concentrate in combination with oat fiber. The resulting meat analogues had a fibrous structure and brown color derived from hempseed protein. They also emphasize the influence of the process parameters on the textural properties of the end product: higher feed moisture content results in decreased hardness, chewiness and cutting strength values, due to the reduced ability of the proteins to cross-link at higher moisture contents, while higher extrusion temperatures resulted in a product with stronger fibers and higher cutting strength. In comparison to soy and pea protein concentrates, hemp protein concentrates show lower water solubility and hydration capacity, but, during the extrusion process, provide extrudates with promising textural properties, which is not the case with hemp protein isolates, probably due to the high concentration of edestin macromolecular complexes, which prevent the formation of fibrous texture [98]. On the other hand, they show good emulsifying activity and emulsion stability index; good foaming capacity; but lowest gelling capacity compared to soy, pea, wheat, buckwheat, fava bean and lupin, and a water holding and fat absorption capacity capacity similar to soy protein isolates [103]. To conclude, hemp seeds can be used in plant-based meat analogues as a source of protein, but also as a source of fat, which, in general, contributes to their economic feasibility. In addition to the presence of numerous amino acids, hemp proteins also have excellent structural properties and, due to their brown color, can also be used as color regulators for the plant-based meat analogues.

Sunflower meal/cake is often referred to as an alternative and sustainable source of protein. Meal/cake is a leftover after sunflower oil production and is mostly used as animal feed, but with novel extraction techniques being developed every day, its proteins have been a topic of many studies. The product is usually considered beneficial because of its neutral taste and lack of allergenicity compared to soy, with a good amino acid profile, but has the downside of containing phenolics such as caffeic and chlorogenic acids, which negatively affect technological and textural properties of sunflower seed proteins [99]. According to Hadidi et al. [104], sunflower seeds contain around 20–25% protein, and sunflower meal/cake contains around 40% protein, which can increase up to 50% after oil removal. From a structural point of view, sunflower proteins are made up of two groups: 11S globulin (helianthinin) and 2S albumin in a 2:1 ratio, but their extraction and isolation is hindered by phenolic compounds, which negatively affect appearance and taste of the extract [105]. To prevent that from happening, phenolics removal is proposed before extraction of proteins with different enzymatic methods, ethanol washing or fermentation [99,100,105]. The application of sunflower proteins for production of plant-based meat analogues was explored by Pöri et al. [99]. This study included fermentation of the sunflower protein concentrate prior to its extrusion using a twin-screw extruder at 250 rpm and different temperature profiles. Extrudates had a fibrous, layered shape and the pre-fermentation had a beneficial effect on their sensory profile. Jia et al. [100] researched the effect of ethanol washing of the sunflower seed cake to produce proteins acceptable for meat analogue production. The samples they produced differed in leftover oil content and phenolic content. They concluded that the removal of phenolics is not mandatory for producing extrudates with favorable properties, but the amount of leftover oil is: de-oiled sunflower seed cake and protein isolates formed a fibrous structure after extrusion at 140 °C, but the ones with oil present did not. In conclusion, although less research has been performed on sunflower seeds in comparison to hemp, mostly due to higher diversity of hemp, sunflower proteins represent an interesting future source of proteins. However, the biggest problem preventing their wider application is the removal of phenolics and oil: prior to their application for plant-based meat analogues, they have to be purified of phenolics and oil, since those two components have been shown to negatively affect the structure of the final products. Up until now, the technologies and methods proposed to purify sunflower proteins are still somewhat too expensive to be applied at a larger scale and they require a lot of energy, water and solvent use.

Rapeseed (*Brassica napus* subsp. *Napus*) is an industrial plant grown primarily because of its high oil contents. Its oldest application is in the production of rapeseed vegetable oil, but in recent years, it has found an increasing application in biodiesel production. The increased need for rapeseed has also resulted in increased amounts of rapeseed meal/cake left over after the production of oil. Rapeseed meal contains about 40% proteins, mainly 12S globulins (cruciferin) and 2S albumins (napin). Similar to sunflower, the greatest drawback of the cake is its richness in glucosinolates, phenolic compounds and phytates [100], which greatly affect the nutritional composition but also the technological properties of the proteins isolated from the meal. Proteins from rapeseed can be used in the form of a concentrate or isolate, both of which have different technological properties. As an example, both rapeseed protein concentrates and isolates are water-insoluble, but the concentrate has higher water holding capacity. Insolubility can occur as a result of oil left over or as a result of surface morphology changes, which can hinder water access during hydration. A 40% *w*/*w* dispersion of a rapeseed protein concentrate showed good rheological properties, which were comparable to those of soy and wheat gluten, which indicated the possibility of its use in meat analogue products [100]. The same group of authors also concluded in another study that a rapeseed protein concentrate forms a fibrous structure in a shear cell at 140 °C, emphasizing its potential use as a protein source in meat analogue production [101].

Besides the above mentioned, there are some studies that explored the properties of other seed proteins such as sesame, chia, nigella, etc. [106,107,108], but the general idea is that all of those oil seeds still need a lot of research to confirm their applicability for meat analogues. Mostly, their application is often hindered by antinutrients, and a negative effect on color and taste of the final product.

### 2.4. Algae as Protein Sources

Algae belongs to the photosynthetic eukaryotes group and can be divided into microalgae and macroalgae (seaweed). Both of those groups show potential in application as protein sources in plant-based meat analogues, with a special focus on microalgae, due to their high biomass yield (15–30 tons annually per unit area) and high production of proteins (around 70% proteins in cells compared to soy, which only has 30–40% proteins in cells) [24]. In comparison to microalgae, protein content of the macroalgal species is lower—according to literature data, red seaweed protein content ranges from 35–47% [109], which is still higher in comparison to other protein sources such as soy, cereals and pseudocereals. Furthermore, the quality of the proteins derived from algae is high: they are rich in essential amino acids [110], with lysine and tryptophan being redundant in all algal species [24]. Seaweed proteins are situated inside the algal cells, and comprise mycosporine-like amino acids, lectins, glycoproteins and phycobiliproteins [110]. Since algae has a cellulose cell wall, a problem of protein extraction arises. The first step in the extraction process has to include rupturing the cell wall (usually mechanically, with ultrasound, or osmotically), and then a proper extraction technique to separate the protein from the rest of the cell contents. Different extraction techniques such as proteolytic-enzyme-assisted extraction, solid–liquid extraction, pulse electric field, high-pressure extraction and microwave- and ultrasound-assisted extraction have been reported to successfully extract proteins [110,111], but the main issue is that most of them do not appear to be economically feasible. Another problem with algae-derived proteins is their poor sensory characteristics—an unacceptable color and a fishy smell—leading to reduced consumer acceptance of such products [112,113].

Techno-functional properties (emulsifying, foaming, gelation and solubility properties) of algal proteins are considered to be in range or even better in comparison to soy [110,114]. Schwenzfeier et al. [115] explored the effect of pH and ionic strength on the foaming properties of algal proteins and concluded that the overall foaming stability of foams made from algal protein isolates is superior to those of whey and albumin in the pH range of 5–7. Caporgno et al. [116] conducted a study in which the aim was to determine how the inclusion of *Auxenochlorella protothecoides* microalgal protein influences the quality of extruded meat analogues. Extrudates produced with the addition of 30% of microalgae at a 60% moisture level had a meat-like fibrous structure and no deleterious effect on color of the extrudates was detected. Furthermore, the incorporation of heterotrophic microalgae in meat analogues also benefits the nutritional composition of the product. In another study by Grahl et al. [117], the effect of *Arthrospira platensis* addition on textural and sensory properties of soy-based extrudates was investigated. High-spirulina-content samples had a dark color, an intense earthy taste and a musty algal odor, and lower textural quality (elasticity, fibrousness and firmness). However, with the optimization of the extrusion process, it was concluded that at low moisture, high temperature and high screw speed, it is possible to partly substitute soy with spirulina to produce meat-like structures. Martinez-Sanz et al. [118] produced foams containing starch and spirulina powders using extrusion cooking with an aim to analyze their micro- and nanostructure. The microstructure of the hybrid foams containing both starch and spirulina had more densely packed and well-connected porous structures, which, in consequence, also affected texture—samples with higher spirulina concentrations exhibited higher hardness. Palanisamy et al. [119] investigated the effect of *Spirulina platensis* flour addition and extrusion parameters on texture of meat analogues and came to a conclusion that spirulina flour addition causes a drop in textural attributes, but this drop can be counterbalanced by the adjustment of extrusion parameters at higher screw speeds and higher water feed. The addition of spirulina improved physical, chemical and nutritional properties of the produced meat analogues.

To sum up, the inclusion of algal proteins in meat analogues requires further investigation. Firstly, new and affordable extraction techniques are needed to procure the proteins, and the production process and composition of the meat analogues has to be strictly optimized so the final product does not have an unwanted color and taste. Color, on one hand, can be regulated by adjustment of the processing parameter, and the taste can be masked using spices. However, another problem that the application of algal proteins is facing is consumer acceptance, which is much lower compared to other protein sources.

## 3. Fats

Besides water, proteins, flavoring, coloring and binding agents, meat alternatives contain between zero and fifteen percent of fats [120]. Combination of the above-mentioned ingredients is responsible for sensorial and textural characteristics of meat alternatives.

Lipid ingredients, such as vegetable oils and fats, improve juiciness, tenderness, mouthfeel and flavor of meat analogues [9,77]. Different types of lipid ingredients (fats/oils) are used during preparation of plant-based meat analogues, with the most common oils being canola, coconut, corn, sunflower and sesame oil, and cocoa butter [78]. Although meat analogues traditionally have low lipid content, their second generation consists of saturated-fat-rich semi-solid constituents (e.g., coconut oil and cocoa butter) [25,70,121,122]. Currently available emulsion-type meat analogues on the market have up to 25% fat content. However, these products are subjected to criticism due to a high amount of saturated fat [78]. Lipids are bioactive components (functional ingredients), and from a health point of view, their inclusion in plant-based meat analogues is of great importance [123]. According to the World Health Organization, dietary fat intake should account for 15% to 30% of total diet energy with cholesterol intake limited to 300 mg/day [124]. Furthermore, nutritionists and dieticians have also recommended limitation of saturated fat and trans-fat intake [125,126,127]. The fatty acid composition of fats or oils derived from plants differs significantly: different vegetable oils contain different quantities of specific fatty acids such as palmitic acid, stearic acid, oleic acid, caprylic acid, capric acid, lauryc acid, myristic acid and linoleic acid [128]. Besides that, plant-based oils/fats include variations in saturated, monounsaturated, polyunsaturated and trans fatty acids, as well as their solid or liquid state [129].

Since abundant consummation of fats is related to a negative impact on human health, researchers are focused on the reduction in actual fat content in meat products [130]. One of the solutions is to introduce fat substitutes, consisting of water and functional ingredients [131,132,133,134]. Fat substitutes could also be used for the preparation of low-fat meat analogues, where textural properties need substantial improvement [25]. Protein particles (from milk, eggs, soy protein isolate), modified (synthetic) lipids and carbohydrates (starch, fibers, cellulose) are already used in the food industry as fat substitutes [129,135,136]. Another example is combination of konjac gels with ingredients such as starch, carrageenan and gellan gum, which can also be utilized for the formulation of low-fat plant-based meat analogues [137]. It is worth noting that fat substitutes have no effect on the sensory properties of meat analogues [25]. However, significant attention should be paid regarding the effect of the oils/fats during preparation of meat analogues with the extrusion process. Oil or fat addition increases juiciness and affects textural properties, such as fiber formation, of the final product [138]. Lipids decrease viscosity of the mixtures used for extrusion and can lubricate the extruder, resulting in a decrease in shearing and friction, and lowering the mechanical energy required to run the extrusion process. This will consequently change the reaction behavior of the proteins, leading to a less fibrous structure. Parameters such as fat/protein interactions, structure formation mechanisms, oil stabilization and component interactions differ for every protein–oil combination and it is difficult to predict the degree of interference of vegetable oils/fats on meat analogue production. Lipids influence the mechanical properties of proteins, as well as the organoleptic perception of the final product [139]. Interactions of fat particles with proteins affect the water-binding capacity, lubricating properties and protein gel strength [140]. Characteristic of the application of oils in plant-based meat products is that the oil needs to be added in the form of droplets. Incorporation depends on the product type and oil type used. If it is very difficult to stabilize incorporating fats, then pre-emulsified oils are used. The advantages of oil pre-emulsion technology include improvement of fat binding ability into a protein matrix and improved oxidative stability of lipids. Additionally, pre-emulsified oils are easier to disperse into water-based systems [141]. Microencapsulation of oils (fish oil emulsions) will facilitate their incorporation in meat analogues, inhibit lipid oxidation, mask undesirable flavors and improve bioavailability of n-3 PUFAs. The amount of oil in this form varies between 1% and 30% while low-molecular-weight compounds are often used as an emulsifier [123]. Structured oils (oleogels) are solid materials, made from fats, which have previously been structurally modified by oleogelators into a three-dimensional thermoreversible gel network. Polymers are often used as structurants, due to the fact that many of them are food-grade and inexpensive. Physical characteristics of edible oleogels enable their use as fat replacers: palm oils, oleins and stearins in combination with additives are mainly used for preparation of meat analogues because they provide desirable textural characteristics. Monoglycerides, lecithin, phytosterols and vegetable waxes are among the most researched structural agents for edible oils. Their combination with other ingredients plays a vital role in the final preparation of plant-based meat analogues [142,143]. An overview of the fats used in plant-based meat production, along with their main textural and structural properties, is summarized in Table 6.

In addition to all of the above, a comprehensive understanding and analysis of lipid sources and their functional roles in plant-based meat analogues, as well as their molecular interactions with other components, are of great importance for the development of the plant-based meat analogue industry [150].

## 4. Structural Ingredients and Stabilizing Agents

For commonly used proteins, which were previously discussed, it is necessary to achieve the desired functionality and acceptability by users [151]; so, addition of stabilizers and structural ingredients is necessary [152]. An exceptional challenge in the preparation of plant alternatives to meat is the extremely large number of ingredients, which is usually over 20 [153], as it can be seen in listed ingredients for vegan/vegetarian bratwurst (Table 7).

Additives that are added in plant-based meat analogues should assure plant-protein functionality through foaming capacity and stability, emulsification, rheology, oil and water holding capacity, gelling and solubility [154]. To ensure functionality and mimic meat, preservatives, stabilizers and dyes, some of which will not be found in meat products, such as lecithin, methylcellulose and titanium dioxide [78,155], are often added to plant-based meat analogues. All added ingredients should assure attributes in accordance with consumer expectations [156].

Meat analogues almost always contain carbohydrates as structural ingredients (as can be seen from Table 4), and they can be in the following forms: starch/flour and gums or binding ingredients [155]. Mattice and Marangoni [153] state that the function of the carbohydrate additives (in the form of starch or flour) is to improve the texture and consistency of the product while the gums/binders will stabilize and shape the product (e.g., methylcellulose, gum acacia, xanthan gum, carrageenan, etc.) [15,156]. There is also an example of using konjac glucomannan in the development of the fermented soybean patty [148], where the produced patty analogue had higher firmness and cohesiveness, and lower stickiness.

The addition of these ingredients enhances the interaction of other components (protein, fat, water) during the preparation process [31,157] and thus the higher carbohydrate content in plant-based meat analogues. At high temperatures, methylcellulose forms a gel network and can create thermo-reversible gel. Addition of a biopolymer to proteins enhances the stability of the mixture needed to form a 3D structure [36]. As presented by Shabazi et al. [36], some of the most frequently used biopolymeric surfactants include the following: (i) ethyl (hydroxyethyl) cellulose stabilizes oil-in-water emulsion through an associate thickening mechanism; (ii) octenyl succinic anhydride attaches to the hydrophilic backbones; (iii) acetylated starch forms a densely packed interface layer on the surface of the oil droplet; and (iv) hydrophobically modified inulin. Recently, there have been significant discussions about the effect of added gums and binders in meat analogues on consumer health [15,151,158]. The focus of recent research is methylcellulose (modified cellulose dietary fiber), which is a very effective binder [159] used also in food packaging and creates a viscous solution (during the digestion), consisting of pseudo-crystalline sequences of trimethyl glucose units [160]. There is a growing trend for using different replacements for methylcellulose as the most commonly used binding ingredient in the plant-based meat analogues. For example, binding performance of methylcellulose hydrogel and a new binder made of pea protein and sugar beet pectin was evaluated on burger meat analogues [161] and bacon-type meat analogues [162], with a conclusion that the homogeneity of the produced analogues was positively affected by the binder addition.

The gums used in the production of meat analogues have not shown any impact on health; however, when purchasing, end users opt for products whose labels indicate environmentally friendly production (such as Eco-Score, Table 4) as well as less processing (NOVA score, Table 4). Precisely because of the above mentioned, efforts are being made to minimize the use of gums as well [156,157,163], which are currently an indispensable ingredient in vegetable meat analogues. Based on literature data, a 2.5% gum arabica/soy protein concentrate can be efficiently used to improve sensory properties of mushroom-based sausage analogues [164]. Study of the effect of different concentrations of a carrageenan, casein, xanthan gum and soy protein concentrate on quality characteristics of mushroom-based sausage analogues showed significant improvement in textural properties of samples prepared with carrageenan [165]. As mentioned before, in plant-based meat analogues, soy and pea proteins are typically the most commonly utilized components. However, those proteins provide poor characteristic juiciness and fibrousness of actual meat. Wheat gluten is also frequently added to the formulations in order to overcome this restriction [166]. Under hydrated conditions, gluten makes plant-based meat mixtures viscous and significantly strengthens plant-based meat mixture adhesion [167]. Besides gluten, there is also research focused on rheological and textural properties of soy-protein-isolate-based meat analogues prepared with guar and xanthan gum, carrageenan, hydroxypropyl and cross-linked tapioca starch as binding agents [168], as well as on organoleptic quality of cowpea protein meat analogues with cocoa pod husk extracts as binding agents [169]. Generally, it can be concluded that, based on the added amount, some ingredients can behave as binders (ingredients with high protein levels), while some can behave as fillers or extenders (ingredients with low or no protein levels) [25].

## 5. Spices

Flavor is one of the essential sensory components of food products [170]. It is important to comprehend the processes involved in the synthesis of flavor molecules in animal flesh to add a meaty flavor to plant-based equivalents [171]. For instance, the flavors of raw and cooked meats change significantly. Raw meat has no aroma and just tastes of blood, metal and salt, while fragrance and flavor compounds in cooked and roasted meat become more complex as processing temperatures rise [172,173]. According to Li and Li [171], flavor compounds in cooked meat are produced with (i) the Maillard reaction between amino acids and reducing sugars; (ii) oxidation of fatty acids; and (iii) thermal degradation of thiamine. The primary ingredients of plant-based meat substitutes are soy protein and wheat gluten, neither of which has the essential intermediate compounds required to give meat-like flavor [155,174]. As a result, to replicate the tastes and scents of meat, several flavorings that resemble meat are added to meat substitutes [175]. To mimic the flavors of processed meat, a wide variety of spices and herbs are added, including those that are also used in the meat processing industry [120]. As previously described by Wang et al. [42], compounds used to improve taste of the plant-based meat analogues can be grouped as follows: (i) natural spices and herbs used to prevent lipid oxidation, (ii) Maillard reaction precursors including reducing sugars, amino acids and thiamine to generate meat-like flavor, (iii) hydrolyzed vegetable proteins used to improve meat-like flavor, (iv) yeast extract contributing to the roasted, meat-like aroma and (v) vegetable oils contributing to mouthfeel of the plant-based meat product. When aromatizing meat analogues, the production process also has to be considered, because analogues that are flavored during extrusion undergo a series of physicochemical changes in the premix [176], and in the case of plant analogues that are produced at a high temperature and/or pressure, the volatile components can significantly change [177]. All of the above greatly change the taste perception, which must remain stable during shelf life [26].

Dried onions, dried garlic, curry powder, black pepper, garlic, chili, paprika and ginger are among the most widely used spice mixtures [120]. According to Vlaic et al. [178], the most widely used aromatic plants in plant-based meat production are parsley, dill, basil, oregano, sage, coriander, rosemary, marjoram, tarragon, bay, thyme and mint. The mentioned herbs contain bioactive molecules that can have a beneficial effect on human health. According to Zioga et al. [179], the addition of herbs and spices masks beany off-flavors, and organic acids, which can be present in those herbs and spices, can also contribute to shelf life. Even yet, a lot of plant-based substitutes still have an aftertaste [180]. As described by Amyoony et al. [181], plant-based meat analogues are characterized by beany and chalky flavors, bitter tastes and “off-flavors” overlapping with unwanted tastes, odors and sensations including astringency that may be brought on by intrinsic components or as a result of storage and processing. The same authors also stated that aftertaste intensity of the plant-based meat analogues was negatively correlated to the overall acceptance of the products based on the sensory evaluation. In addition, vegetable oils, which can influence taste, are combined with the protein components to simulate mouthfeel and juiciness. This applies in particular to coconut oil, which can be mixed with liquid oils such as sunflower and rapeseed oil [182].

The distinct beany smell, which is believed to be associated with the byproducts or derivatives of secondary lipid oxidation, including hexanal and methanethiol, along with the naturally occurring bitter-astringent tastes resulting from saponins and isoflavones, may pose a challenge for the use of soy protein as a building block for meat substitutes [56,183].

Yuan et al. [184] analyzed the influence of four selected spices (black pepper, red pepper, onion and garlic) on the flavor of 50 commercial meat analogues. According to their findings, incorporating those spices into the extrusion process decreased the production of certain volatile chemicals linked to heat-related treatments. So, in addition to improving flavor profiles of extrudates, adding spices can delay the production of some volatile chemicals with bad flavors, such as alcohols, ketones and aldehydes. Similarly, Yuan et al. [185] used a five-spice powder mixture in the production of a mushroom-based meat sausage analogue. They analyzed the effect of the water content, type of mushroom and amount of added powder on physicochemical and structural properties and the aroma profile of prepared meat analogues. They successfully produced a plant-based meat analogue whose aroma was close to that of beef and concluded that, besides the spice mixture used in the formulation, the aroma profile is governed by volatile flavor substances, which are mostly a result of the complexities of lipid oxidation and bacterial metabolism.

Similar to other constituents of plant-based meat products, spices also have the ability to affect structural properties of the mixtures, and, once again, the complexity of their interactions with other components (proteins, fats, stabilizers) is still an area where much research is needed.

## 6. Coloring Agents

Color is one of the most important factors. Also, hydrated alginate and maltodextrin are coloring agents that reduce color migration during production, which governs consumers’ product acceptance [186]. Generally, raw fresh meat has a red color, which turns to brown during cooking and it is a challenge to obtain plant-based meat to mimic and resemble traditional meat. Gluten and soy, as some of the most widely used plant-based proteins, are originally yellow or beige in nature, which raises the need to improve their color to be more similar to that of meat, by using coloring ingredients or precursor substances [187].

Consequently, natural pigments have been developed [188,189] but they cannot be used directly from renewable sources because the incorporation of raw materials as coloring agents has many limitations (e.g., natural pigments are unstable at a high pressure and high temperature, chemically degrade when subjected to oxygen and lose their acceptance and functionality during storage) [188]. Nevertheless, natural colorants have remarkable antioxidant properties, suggesting that they could be used as nitrite replacers in meat products, as well as flavor and textural property enhancers [182,188]. The simulation of the color of cooked/roasted meat is achieved by using caramel colors (annatto or malt; carotene, cumin, turmin) [190] and beet root extracts [191], but also by adding reducing sugars (e.g., dextrose, maltose, lactose, xylose, galactose, mannose and arabinose). Reducing sugars can react with amine groups in proteins (Maillard reaction) [192,193]. In addition, color additives can also be used to impart a reddish-brown color. One of the color additives described is soy leghemoglobin, which was approved by the Food and Drug Administration in 2019 [194].

In the production of plant-based meat products, the thermal stability and pH sensitivity are of great importance. The degradation of thermally unstable agents can lead to an unacceptable color appearance [187]. To ensure stability, juices rich in polyphenols and ascorbic acid (apple extracts or citrus fruit extracts) are often added to plant-based meat products [15,195]. Furthermore, juices can also act as antimicrobial and preservative agents [195]. Other examples of coloring agents include carotene, cumin and turmin, which are considered heat-stable and preferred by consumers [196]. The red meat color can also be mimicked by leghemoglobin, lycopene, annatto and beet juice extracts, while chicken meat color can be mimicked by titanium dioxide [15]. As mentioned earlier, coloring agents can be combined with proteins before the structuring treatment or mixed with the semi-structured plant-based materials during structuring [187]. Also, hydrated alginate and maltodextrin are coloring agents that reduce color migration during the production [187].

Ryu et al. [186] investigated the use of different natural pigments in plant-based meat to imitate the color of meat after cooking and the results confirmed that natural pigments can be used as alternative colorants in plant-based meat. Also, Bakhsh et al. [197] applied natural colorants such as lactoferrin and red yeast rice to plant-based patties and their results showed that the use of natural colorants had a negligible effect on the chemical composition and textural attributes, and the colors of the products were lighter and had a brownish tinge in comparison to the control. A similar investigation was carried out by Akramzadeh et al. [198], where the authors analyzed three different natural color agents (red yeast rice, lycopene and paprika oleoresin) used as ingredients in non-meat sausages. Respectively, the products provided superior sensory acceptance.

## 7. Conclusions

Plant-based meat analogue production is on the rise. It is mainly driven by an ever-rising awareness of climate change and the need for the food industry to reduce CO_2_ emissions. Plant-based food holds the promise toward a cleaner and healthier future, from one side due to the reduction in the environmental impact, and, on the other side, because plant-based foods are known to possess many beneficial effects. However, the biggest problem that still remains to be solved is the difference in texture, mouthfeel and taste of the plant-based meat analogues in comparison to real meat. This review paper has shown that there is an abundance of literature available on the topic of texture and structure of plant-based meat analogues, but somehow, a majority of literature data come to the same conclusion: the interactions of particular components of plant-based meat analogues are extremely complex and highly dependent on the processing conditions during production and, subsequently, the processing (cooking, baking, etc.) conditions at home, after the product has been purchased by the consumer. Therefore, the need for optimization of the composition of the mixtures, as well as the processing conditions, has to be highly stressed. In general, the development of plant-based meat analogues is a complex work that requires the expertise of many different specialists—from nutritionists who can define optimal nutritive content and food technologists who can optimize the structural properties of the analogue to engineers who can adapt the manufacturing process to specific needs.

## Figures and Tables

**Table 1:** Compounds present in soybean with their functional properties.

Group	Compounds	Functionality	Reference
Proteins	β-conglycinin glycinin storage proteins lecithin Bowman–Birk (BBI) protease inhibitors	Cholesterol-lowering, body fat reduction, reducing the risk of coronary diseases; strong texture regulating properties	[21]
Bioactive peptides	numerous	Usually inactive, activate during processing or ingestion, fast absorption in the GI tract, anti-diabetic, anti-hypertensive, anti-cancer	[22]
Isoflavones	glycosides: genistin, diadzin, glycetin (inactive form); aglycones: genistein, diadzein and glycetein (bioactive form)	Inactive prior to digestion; heart disease, diabetes, menopausal symptoms, osteoporosis and prostate and breast cancer prevention	[20]
Saponins	triterpenoic saponins	Anti-inflammatory, anti-carcinogenic, antimicrobial, hepato- and cardio-protective effects	[20,23]

**Table 2:** An overview of the most recent literature on the application of pea proteins in meat-based analogue production.

Protein	Process	Textural Properties	Reference
Pea protein isolate (PPI) + wheat gluten	High temperature shear, 95–140 °C	Fibrous structures at 120 °C, lower processing temperatures resulted in low tensile strength, temperatures higher than 120 °C gave a strong and layered product; matrix strength similar to chicken meat	[38]
Pea protein isolate + amylose/amylopectin mixtures	High-moisture extrusion, 60–120 °C	Exploration of the interactions between starch and pea proteins—amylopectin contributes to viscosity and fibrousness, amylose does not	[41]
Pea protein isolate + maize starch + soy lecithin + beef fat	Large volume extrusion (LVE) 3D printer, printing speed at 15 mm/s, 100% infill density, two nozzle sizes (1.54 and 2.16 mm)	PPI + starch paste showed weak gel behavior, increased viscosity due to starch addition; optimum printing nozzle with a 1.54 mm diameter and 15 mm/s printing speed	[42]
Pea protein concentrate	Extrusion at 140 °C, 400 rpm screw speed, 3.6 kg/h water supply	Layered structures, chewiness higher in comparison to rice protein extrudates, pea protein forms intermolecular hydrogen bonds during extrusion	[35]
Pea protein isolate dispersions (10–21% *w*/*w*)	Three-dimensional extrusion printing, nozzle diameter of 1.6 mm, room temperature	At higher PPI concentrations (>17% *w*/*w*), the paste strength increased and PPI produced stable 3D-printed shapes; however, too high PPI concentrations lead to uneven extrusion, an inhomogeneity in the surface structure of the 3D-printed object	[43]
Pea protein concentrate	Extraction of globulin proteins responsible for forming fragile gels followed by high-moisture extrusion in a twin-screw extruder at 200 rpm, 30–125 °C, 42% dry matter, 12 kg/h of wet feed	Pea-soluble protein extracts had higher gelation capacity compared to powdered protein isolates. Purified protein isolates have an enhancing effect on gel elasticity and reduce brittleness	[44]
Isolated pea protein	Intermediate moisture (50%) twin screw extrusion, 250 rpm, 100–150 °C	Sponge-like structure, rich in essential amino acids, good oil absorption and emulsion properties	[32]
Pea protein isolates	High-moisture-extrusion cooking, 100–160 °C cooking temperature, 0.45 kg/h dry protein feed rate, water to regulate moisture of the mix at 55%	Fiber formation and texture properties can be controlled by the cooking temperature, feed powders with larger particles are easier to process	[45]

**Table 3:** An overview of the most recent literature on the application of chickpeas, lentils, beans and peanuts in meat-based analogue production.

Protein Source	Structural and Textural Characteristics	Reference
Chickpea	Low foaming capacity in comparison to soy, high foam stability, gelling ability similar to that of soy, high water and oil binding capacity, which is beneficial for use in meat analogues, can also be used as colorants	[48,49]
Lentils	Gelling capability comparable to whey proteins, but highly pH dependent, oil holding and foaming capacity comparable to soy, excellent emulsifying characteristics, high gel strength	[50,51,52]
Faba beans	Heat treatment and low-moisture extrusion cause a rise in water holding capacity, water solubility and gel strength, fibrous layered structure can be obtained with high-moisture extrusion	[53,54,55]
Mung beans	Albumins have better textural stability, texturization properties are temperature dependent	[56,57]
Peanuts	Arachin is the main protein that changes during extrusion forming layered structures, in combination with other ingredients (e.g., carrageenan, gellan gum, transglutaminase) giving increased gel strength, storage modulus and fracture stress	[58,59,60]

**Table 4:** Proximate protein contents of cereals used in plant-based meat analogue production [77].

Cereal	Protein Content (% Dry Matter)
Wheat (*Triticum aestivum*)	8–17.5
Maize (*Zea mays*)	8.8–11.9
Rice (*Oryza sativa*)	7–10
Oats (*Avena sativa*)	8.7–16

**Table 5:** Structural and textural characteristics of some oil seed proteins used in plant-based meat production.

Oil Seed Protein Source	Structural and Textural Characteristics	Reference
Industrial hemp seed	Globulins and albumins with β-sheets give fibrous-like structures during extrusion, with a significant influence of process conditions (high moisture does not favor textural properties, while increased temperature does); low water solubility, good emulsifying and gelling properties, foaming, water and fat holding capacity similar to soy protein isolates	[95,96,97,98]
Sunflower meal/cake	Pre-fermented sunflower extrudates form fibrous, layered structures during extrusion, it was found that de-fatting is required to obtain a fibrous structure of the extrudates	[99,100]
Rapeseed	Rapeseed protein concentrate has better solubility in comparison to isolate, fibrous structure formation in a shear cell at 140 °C	[101]

**Table 6:** An overview of the most recent literature on the application of lipid ingredients (fats/oils) in meat-based analogue production.

Fats/Oils	Specific Fatty Acid Composition	Textural Properties	Reference
Sunflower oil	Palmitic acid (C16:0), stearic acid (C18:0), oleic acid (C18:1), linoleic acid (C18:2)	Meat analogues based on sunflower protein concentrate (SP), fermented sunflower protein concentrate (fSP) and fSP with pH shifted to neutral (fSP7) prepared with high-moisture extrusion; fermentation, in combination with neutral pH shift, enabled formation of fibrillar structures.	[99,128]
Canola oil	Palmitic acid (C16:0), stearic acid (C18:0), oleic acid (C18:1), linoleic acid (C18:2), linolenic acid (C18:3)	Canola oil, together with textured vegetable protein (TVP), formed a compact protein gel network of TVP matrix; hydrophobic interactions between the oil globule and the amino acids in protein resulted in a firmer meat product.	[128,144]
Coconut oil	Caprylic acid (C8:0), capric acid (C10:0), lauric acid (C12:0), myristic acid (C14:0), palmitic acid (C16:0), stearic acid (C18:0), oleic acid (C18:1), linoleic acid (C18:2)	Development of 3D-printable emulsified fat analogues using konjac glucomannan and coconut oil: before cooking, the printed fat analogues showed acceptable shape stability and surface smoothness. Significant oil release of fat analogues occurred after cooking. Additionally, higher coconut oil content added to the fat analogues led to the release of a larger oil amount and lower hardness and tensile strength after cooking.	[128,145]
Palm kernel oil	Lauric acid (C12:0), palmitic acid (C16:0), stearic acid (C18:0), oleic acid (C18:1), palmitoleic acid (C16:1), eicosenoic acid (C20:1), linoleic acid (C18:2), linolenic acid (C18:3)	For partial replacement of hydrogenated fats, an increasing concern raised due to high content of saturated fatty acids. On the other hand, palm oil exhibits potential to improve retention and succulence of meat analogues.	[128,142,146]
Peanut oil	Valeric acid (C4:0), caprylic acid (C8:0), decanoic acid (C10:0), lauric acid (C12:0), myristic acid (C14:0), palmitic acid (C16:0), stearic acid (C18:0), palmitoleic acid (C16:1), eicosenoic acid (C20:1), oleic acid (C18:1), erucic acid (C22:1), linoleic acid (C18:2), linolenic acid (C18:3), arachidonic acid (C20:4), eicosapentaenoic acid (C20:5 n-3 EPA)	For the production of oleogels; addition of higher amounts of beeswax leads to increased elasticity, macroscopic viscosity and firmness values of oleogels. Formation of structured network was more pronounced.	[128,147]
Rapeseed oil	Saturated (unknown), monounsaturated (unknown), polyunsaturated (unknown)	The presence of rapeseed oil, in combination with pea protein and soy protein, decreased gel strength, Young’s modulus and the length of the LVE region; rapeseed oil droplets were not bound to the protein matrix; higher amount of extracted oil decreased encapsulation efficiency of pea protein.	[128,139]
Soybean oil	Palmitic acid (C16:0), stearic acid (C18:0), oleic acid (C18:1), linoleic acid (C18:2), linolenic acid (C18:3)	Soybean is commonly used as a source of plant protein and lipids; soybean in combination with konjac glucomannan improves stickiness, cohesiveness and firmness of meat analogue.	[128,148]
Sesame oil	Palmitic acid (C16:0), stearic acid (C18:0), oleic acid (C18:1), palmitoleic acid (C16:1), eicosenoic acid (C20:1), linoleic acid (C18:2), linolenic acid (C18:3)	Use of sesame oil for oleogel formation to replace animal fats in beef burger; the addition of higher beeswax amount reduced oleogel hardness and consequently decreased the hardness, gumminess and chewiness of the raw burgers.	[128,149]
Cocoa butter	Palmitic acid (C16:0), stearic acid (C18:0), oleic acid (C18:1)	Three-dimensional printing fluidity and formability of the soy-protein- and wheat-gluten-based materials were promoted with the addition of thermosensitive cocoa butter.	[81,128]

**Table 7:** Ingredients and nutrition facts of veggie and bio bratwurst.

Facts	Veggie Fresh Bratwurst ^1^	Bio Bratwurst ^2^	Veggie Mini Bratwurst ^3^
Ingredients	Tofu (soybeans, water, coagulant: magnesium, chloride (nigari)), water, wheat protein, sunflower oil, sea salt, spices, row cane sugar, celery, thickener: locust bean gum	Organic pork (98%), sea salt, spices, dextrose, antioxidant: ascorbic acid; herbs, natural casing (sheep)	Water, pea protein isolate, onion cubes, rapeseed oil, coconut fat, textured pea protein (pea protein, pea flour), brandy vinegar, wheat gluten, thickener: methyl cellulose, apple cider vinegar, citrus fiber, pea fiber, gluten-free full oat grain flour, spices, spice extracts, table salt, coloring vegetable concentrate (carrot, beetroot), yeast extract, natural flavor, stabilizers: sodium alginate, konjac, guar gum
Allergens	Celery, gluten, soybeans		Gluten
Energy, kJ (kcal)	979 (234)	991 (237)	799 (191)
Fat (g)	13	18	12
Saturated fat (g)	1.6	6.9	5.4
Carbohydrates (g)	2.9	0.5	2.1
Sugars (g)	2.7	0.5	1
Proteins (g)	26	18	16
Salt (g)	1.9	1.6	1.6
Nutri-score *	D	D	D
NOVA ^#^	4	4	4
Eco-Score ^$^	B	Unknown	A

*—the nutritional quality of food products with A (highest value) to E grades (lowest value); ^#^—food product classification according to their degree of processing; (1) unprocessed or minimally processed foods, (2) processed culinary ingredients, (3) processed foods and (4) ultra processed foods; ^$^—summarized environmental impacts of food products with A to E grades. ^1^: https://world.openfoodfacts.org/product/4019738031497/veggiefresh-bratwurst-viana#panel_nutriscore; accessed on 26 October 2023 ^2^: https://world.openfoodfacts.org/product/4061458025201/bio-bratwurst-vom-schwein-gut-bio; accessed on 26 October 2023 ^3^: https://world.openfoodfacts.org/product/4251349104171/veggie-mini-bratwurst-amidori. accessed on 26 October 2023.
